# Ethyl 4-(2-chloro­quinolin-3-yl)-1-phenyl-1*H*-pyrrole-3-carboxyl­ate

**DOI:** 10.1107/S1600536808032546

**Published:** 2008-10-15

**Authors:** Saida Benzerka, Abdelmalek Bouraiou, Sofiane Bouacida, Abdelmadjid Debache, Ali Belfaitah

**Affiliations:** a Laboratoire des Produits Naturels d’Origine Végétale et de Synthèse Organique, PHYSYNOR, Université Mentouri-Constantine, 25000 Constantine, Algeria; b Laboratoire de Chimie Moléculaire, du Contrôle de l’Environnement et de Mesures Physico-Chimiques, Faculté des Sciences, Département de Chimie, Université Mentouri, 25000 Constantine, Algeria; c Département de Chimie, Faculté des Sciences et Sciences de l’Ingénieur, Université A.Mira de Béjaia, Route Targua Ouzmour 06000 Béjaia, Algeria

## Abstract

In the mol­ecule of the title compound, C_22_H_17_ClN_2_O_2_, the dihedral angles formed by the pyrrole ring with the quinoline and phenyl rings are 67.93 (8) and 28.40 (11)°, respectively. In the crystal structure, mol­ecules are linked into dimers by inter­molecular C—H⋯O hydrogen bonds.

## Related literature

For general background, see: Corvo & Pereira (2002 ▶); Harrison *et al.* (2006 ▶); Wright *et al.* (2001 ▶); Sahu *et al.* (2002 ▶); Michael (1997 ▶); Rezig *et al.* (2000 ▶); Raj Amal *et al.* (2003 ▶); Witherup *et al.* (1995 ▶); Moussaoui *et al.* (2002 ▶). For related structures, see: Belfaitah *et al.* (2006 ▶); Bouraiou *et al.* (2008 ▶);. For details of the synthesis, see: Menasra *et al.* (2005 ▶); Benzerka *et al.* (2008 ▶). For pyrroles as building blocks in naturally occurring and biologically active compounds such as heme, chloro­phyll and vitamin B12, see: Bigg & Bonnaud (1994 ▶); Demir *et al.* (2005 ▶); Tsukamoto *et al.* (2001 ▶);
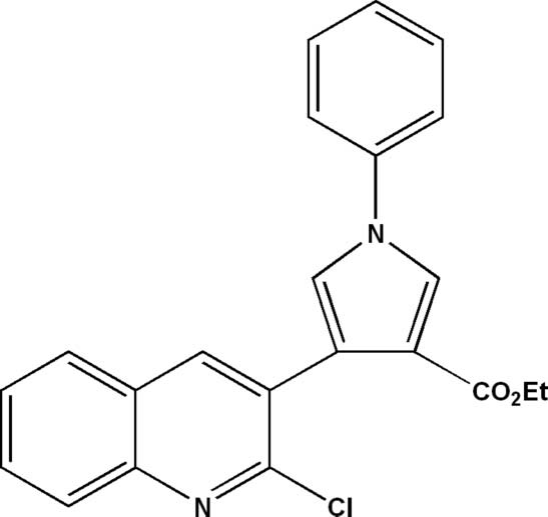


## Experimental

### 

#### Crystal data

C_22_H_17_ClN_2_O_2_
*M* *_r_* = 376.83
Monoclinic, 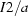

*a* = 20.2021 (6) Å
*b* = 8.0500 (1) Å
*c* = 24.0238 (7) Å
β = 105.29 (2)°
*V* = 3768.6 (4) Å^3^
*Z* = 8
Mo *K*α radiation
μ = 0.22 mm^−1^
*T* = 296 (2) K
0.15 × 0.06 × 0.05 mm

#### Data collection

Nonius KappaCCD diffractometer
Absorption correction: none
9812 measured reflections
3315 independent reflections
2343 reflections with *I* > 2σ(*I*)
*R*_int_ = 0.043

#### Refinement

*R*[*F* ^2^ > 2σ(*F* ^2^)] = 0.051
*wR*(*F* ^2^) = 0.142
*S* = 1.02
3315 reflections
244 parameters
H-atom parameters constrained
Δρ_max_ = 0.29 e Å^−3^
Δρ_min_ = −0.27 e Å^−3^

### 

Data collection: *COLLECT* (Nonius, 1998 ▶); cell refinement: *DENZO*/*SCALEPACK* (Otwinowski & Minor, 1997 ▶); data reduction: *DENZO*/*SCALEPACK*; program(s) used to solve structure: *SIR2002* (Burla *et al.*, 2003 ▶); program(s) used to refine structure: *SHELXL97* (Sheldrick, 2008 ▶); molecular graphics: *ORTEP-3* (Farrugia, 1997 ▶) and *DIAMOND* (Brandenburg & Berndt, 2001 ▶); software used to prepare material for publication: *WinGX* (Farrugia, 1999 ▶).

## Supplementary Material

Crystal structure: contains datablocks global, I. DOI: 10.1107/S1600536808032546/rz2251sup1.cif

Structure factors: contains datablocks I. DOI: 10.1107/S1600536808032546/rz2251Isup2.hkl

Additional supplementary materials:  crystallographic information; 3D view; checkCIF report

## Figures and Tables

**Table 1:** Hydrogen-bond geometry (Å, °)

*D*—H⋯*A*	*D*—H	H⋯*A*	*D*⋯*A*	*D*—H⋯*A*
C12—H12⋯O1^i^	0.93	2.50	3.383 (3)	159
C15—H15⋯O1^i^	0.93	2.45	3.275 (4)	148

Symmetry code: (i) 
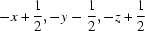
.

**Fig. 1.**
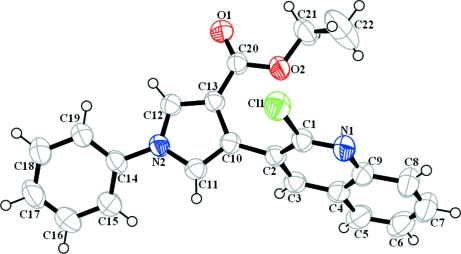
The molecular structure of the title compound with the atomic labelling scheme. Displacement ellipsoids are drawn at the 50% probability level.

**Fig. 2.**
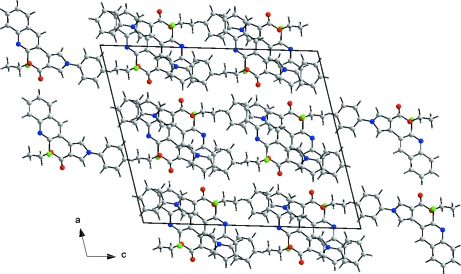
Packing diagram of the title compound viewed along the b axis.

## supplementary crystallographic information

### Comment

Heterocyclic compounds and particularly five and six membered ring compounds occupy a prominent place among various classes of organic compounds for their diverse biological activities. Among a wide variety of heterocycles that have been explored for developing pharmaceutically important molecules (Raj Amal *et al.*, 2003), quinolines have played an important role in medicine chemistry (Wright *et al.*, 2001; Sahu *et al.*, 2002). Some of them have received considerable attention due to their presence in numerous natural products along with their wide ranging application as drugs, pharmaceutical and agrochemicals (Michael, 1997). Pyrroles are an important class of heterocyclic compounds and are widely used in synthetic organic chemistry and material science (Corvo & Pereira, 2002; Harrison *et al.*, 2006). Pyrroles are often seen as building blocks in naturally occurring and biologically active compounds such as heme, chlorophyll and vitamin B12 (Demir *et al.*, 2005; Bigg & Bonnaud, 1994; Tsukamoto *et al.*, 2001). Syntheses of pyrroloquinolines derivatives were not particularly numerous in the literature before the initial report of the unique alkaloids (Witherup *et al.*, 1995). In a continuation of our program on the synthesis and biological evaluation of quinolines derivatives (Belfaitah *et al.*, 2006; Bouraiou *et al.*, 2008; Rezig *et al.*, 2000; Moussaoui *et al.*, 2002), we have elaborated an efficient route for the synthesis of 3-pyrrolylquinolines by dehydrogenation of 3-pyrrolidinyl quinolines using activated manganese dioxide in refluxing THF during 5 h (Menasra *et al.*, 2005; Benzerka *et al.* 2008). We report here the crystal structure of a new *N*-phenylpyrrole derivative bearing a quinoline ring at C-3 and an ester group at C-4.

The molecular structure and the atom-numbering scheme of the title compound are shown in Fig. 1. The quinoline ring system is essentially planar, the dihedral angle formed by the six-membered rings being only 0.64 (8)°. The dihedral angles between the pyrrole ring and the quinoline and phenyl rings are 67.93 (8) and 28.40 (11)°, respectively. The geometric parameters are in agreement with those of other structures possessing a quinolyl substituent previously reported in the literature (Belfaitah *et al.*, 2006; Bouraiou *et al.*, 2008). In the crystal structure (Fig. 2), molecules are linked into dimers by intermolecular C—H···O hydrogen bonds (Table 1).

### Experimental

To a 0.1 *M* solution of ethyl 4-(2-chloroquinolin-3-yl)-1-phenylpyrrole -3-carboxylate (1.5 mmol) in dry THF (15 ml) 2.5 equivalents of activated MnO_2_ were added. The mixture was refluxed for 2.5 h. The same amount of activated MnO_2_ was then added, and the reflux was continued for an additional 2.5 h. After cooling, the mixture was diluted with THF, filtered through Celite and washed with THF (5x5 ml). The filtrate was concentrated under reduced pressure, diluted with CH_2_Cl_2_ and washed with water (2x5 ml). The organic layers were separated and dried over anhydrous MgSO_4_. The filtrate was concentrated and the residue was purified by flash chromatography on silica gel using AcOEt/pentane (2:1 v/v) as eluent to afford the corresponding pure pyrrole derivative. Crystals suitable for X-ray analysis were obtained by slow evaporation of the solvent.

### Refinement

All H atoms were introduced in calculated positions and treated as riding, with C—H = 0.93-0.97 Å, and with *U*_iso_(H) = 1.2 *U*_eq_(C) or 1.5 *U*_eq_(C) for methyl hydrogen atoms.

### Crystal data

**Table:**

C_22_H_17_ClN_2_O_2_	*F*(000) = 1568
*M**_r_* = 376.83	*D*_x_ = 1.328 Mg m^−^^3^
Monoclinic, *I*2/*a*	Mo *K*α radiation, λ = 0.71073 Å
Hall symbol: -I 2ya	Cell parameters from 9812 reflections
*a* = 20.2021 (6) Å	θ = 5.1–25.1°
*b* = 8.0500 (1) Å	µ = 0.22 mm^−^^1^
*c* = 24.0238 (7) Å	*T* = 296 K
β = 105.29 (2)°	Needle, white
*V* = 3768.6 (4) Å^3^	0.15 × 0.06 × 0.05 mm
*Z* = 8	

### Data collection

**Table:**

Nonius KappaCCD diffractometer	2343 reflections with *I* > 2σ(*I*)
Radiation source: fine-focus sealed tube	*R*_int_ = 0.043
graphite	θ_max_ = 25.1°, θ_min_ = 5.1°
φ scans, and ω scans with κ offsets	*h* = −23→24
9812 measured reflections	*k* = −9→9
3315 independent reflections	*l* = −28→28

### Refinement

**Table:**

Refinement on *F*^2^	Primary atom site location: structure-invariant direct methods
Least-squares matrix: full	Secondary atom site location: difference Fourier map
*R*[*F*^2^ > 2σ(*F*^2^)] = 0.051	Hydrogen site location: inferred from neighbouring sites
*wR*(*F*^2^) = 0.142	H-atom parameters constrained
*S* = 1.02	*w* = 1/[σ^2^(*F*_o_^2^) + (0.103*P*)^2^ + 0.3358*P*] where *P* = (*F*_o_^2^ + 2*F*_c_^2^)/3
3315 reflections	(Δ/σ)_max_ < 0.001
244 parameters	Δρ_max_ = 0.29 e Å^−^^3^
0 restraints	Δρ_min_ = −0.27 e Å^−^^3^

### Special details

**Table:**

Geometry. All e.s.d.'s (except the e.s.d. in the dihedral angle between two l.s. planes) are estimated using the full covariance matrix. The cell e.s.d.'s are taken into account individually in the estimation of e.s.d.'s in distances, angles and torsion angles; correlations between e.s.d.'s in cell parameters are only used when they are defined by crystal symmetry. An approximate (isotropic) treatment of cell e.s.d.'s is used for estimating e.s.d.'s involving l.s. planes.
Refinement. Refinement of *F*^2^ against ALL reflections. The weighted *R*-factor *wR* and goodness of fit *S* are based on *F*^2^, conventional *R*-factors *R* are based on *F*, with *F* set to zero for negative *F*^2^. The threshold expression of *F*^2^ > σ(*F*^2^) is used only for calculating *R*-factors(gt) *etc*. and is not relevant to the choice of reflections for refinement. *R*-factors based on *F*^2^ are statistically about twice as large as those based on *F*, and *R*- factors based on ALL data will be even larger.

### Fractional atomic coordinates and isotropic or equivalent isotropic displacement parameters (Å^2^)

**Table:**

	*x*	*y*	*z*	*U*_iso_*/*U*_eq_	
Cl1	0.12476 (3)	0.20796 (10)	0.35827 (3)	0.0669 (3)	
O1	0.19940 (10)	−0.3023 (3)	0.31308 (8)	0.0722 (6)	
C13	0.12321 (12)	−0.1022 (3)	0.25970 (10)	0.0454 (6)	
N2	0.10945 (10)	0.0124 (3)	0.17336 (8)	0.0480 (5)	
N1	0.00661 (12)	0.1516 (3)	0.37746 (9)	0.0607 (6)	
C3	−0.04742 (13)	−0.0147 (3)	0.27328 (11)	0.0515 (6)	
H3	−0.0658	−0.0671	0.2381	0.062*	
O2	0.11506 (10)	−0.2065 (3)	0.34790 (8)	0.0696 (6)	
C20	0.15071 (12)	−0.2127 (3)	0.30819 (10)	0.0489 (6)	
C12	0.14907 (12)	−0.0931 (3)	0.21243 (10)	0.0483 (6)	
H12	0.1874	−0.1498	0.2079	0.058*	
C1	0.04143 (13)	0.1247 (3)	0.34028 (10)	0.0509 (6)	
C14	0.12066 (13)	0.0599 (3)	0.11921 (10)	0.0507 (6)	
C10	0.06434 (12)	0.0057 (3)	0.24895 (10)	0.0445 (5)	
C11	0.05718 (12)	0.0714 (3)	0.19565 (10)	0.0490 (6)	
H11	0.0227	0.1444	0.1770	0.059*	
C2	0.01905 (12)	0.0392 (3)	0.28691 (10)	0.0455 (6)	
C4	−0.08852 (13)	0.0083 (3)	0.31192 (12)	0.0555 (7)	
C9	−0.05921 (14)	0.0900 (4)	0.36430 (12)	0.0601 (7)	
C5	−0.15703 (14)	−0.0503 (4)	0.30059 (15)	0.0705 (8)	
H5	−0.1772	−0.1042	0.2660	0.085*	
C16	0.19620 (18)	0.1089 (5)	0.05969 (13)	0.0772 (9)	
H16	0.2402	0.1075	0.0546	0.093*	
C15	0.18577 (15)	0.0595 (4)	0.11161 (12)	0.0635 (7)	
H15	0.2228	0.0258	0.1415	0.076*	
C7	−0.1635 (2)	0.0529 (5)	0.39232 (18)	0.0898 (11)	
H7	−0.1889	0.0675	0.4190	0.108*	
C6	−0.19327 (17)	−0.0274 (4)	0.34045 (18)	0.0827 (10)	
H6	−0.2382	−0.0658	0.3330	0.099*	
C8	−0.09838 (17)	0.1100 (5)	0.40466 (15)	0.0802 (10)	
H8	−0.0793	0.1626	0.4397	0.096*	
C19	0.06632 (17)	0.1077 (5)	0.07449 (12)	0.0823 (10)	
H19	0.0219	0.1053	0.0788	0.099*	
C17	0.14196 (19)	0.1601 (5)	0.01550 (13)	0.0817 (10)	
H17	0.1492	0.1950	−0.0193	0.098*	
C21	0.1396 (2)	−0.3041 (5)	0.39981 (13)	0.0838 (10)	
H21A	0.1874	−0.3336	0.4046	0.101*	
H21B	0.1132	−0.4057	0.3970	0.101*	
C18	0.07782 (19)	0.1593 (6)	0.02294 (13)	0.0936 (12)	
H18	0.0410	0.1940	−0.0070	0.112*	
C22	0.1328 (3)	−0.2075 (6)	0.44902 (17)	0.141 (2)	
H22A	0.1489	−0.2719	0.4836	0.211*	
H22B	0.0855	−0.1791	0.4441	0.211*	
H22C	0.1596	−0.1078	0.4519	0.211*	

### Atomic displacement parameters (Å^2^)

**Table:**

	*U*^11^	*U*^22^	*U*^33^	*U*^12^	*U*^13^	*U*^23^
Cl1	0.0655 (4)	0.0788 (5)	0.0550 (4)	−0.0096 (4)	0.0136 (3)	−0.0156 (3)
O1	0.0693 (12)	0.0869 (15)	0.0660 (12)	0.0322 (12)	0.0276 (10)	0.0229 (11)
C13	0.0496 (12)	0.0459 (14)	0.0408 (12)	0.0020 (11)	0.0120 (10)	−0.0005 (10)
N2	0.0535 (11)	0.0519 (12)	0.0399 (10)	0.0034 (10)	0.0144 (9)	0.0006 (9)
N1	0.0674 (14)	0.0676 (15)	0.0516 (12)	0.0092 (12)	0.0239 (11)	−0.0019 (11)
C3	0.0547 (14)	0.0473 (15)	0.0517 (14)	0.0051 (11)	0.0127 (11)	0.0025 (11)
O2	0.0821 (13)	0.0810 (14)	0.0542 (11)	0.0318 (11)	0.0330 (10)	0.0230 (10)
C20	0.0540 (14)	0.0486 (14)	0.0461 (13)	0.0043 (12)	0.0170 (11)	0.0016 (11)
C12	0.0521 (13)	0.0487 (15)	0.0456 (13)	0.0050 (11)	0.0154 (11)	0.0002 (11)
C1	0.0578 (14)	0.0514 (15)	0.0443 (13)	0.0036 (12)	0.0153 (11)	−0.0017 (11)
C14	0.0635 (15)	0.0514 (15)	0.0379 (12)	0.0035 (12)	0.0143 (11)	0.0013 (11)
C10	0.0483 (12)	0.0434 (13)	0.0413 (12)	0.0015 (11)	0.0112 (10)	−0.0039 (10)
C11	0.0502 (13)	0.0497 (14)	0.0466 (13)	0.0060 (11)	0.0116 (10)	−0.0004 (11)
C2	0.0512 (13)	0.0424 (14)	0.0428 (13)	0.0056 (10)	0.0124 (10)	0.0010 (10)
C4	0.0578 (15)	0.0498 (15)	0.0622 (16)	0.0104 (12)	0.0219 (12)	0.0127 (13)
C9	0.0659 (16)	0.0611 (18)	0.0589 (16)	0.0149 (14)	0.0262 (13)	0.0082 (13)
C5	0.0592 (16)	0.0652 (19)	0.091 (2)	0.0067 (14)	0.0263 (16)	0.0113 (16)
C16	0.088 (2)	0.093 (2)	0.0613 (18)	0.0162 (19)	0.0381 (16)	0.0154 (17)
C15	0.0694 (17)	0.0738 (19)	0.0515 (15)	0.0134 (15)	0.0235 (13)	0.0112 (14)
C7	0.089 (2)	0.103 (3)	0.095 (3)	0.020 (2)	0.054 (2)	0.016 (2)
C6	0.0617 (17)	0.078 (2)	0.117 (3)	0.0093 (17)	0.0390 (19)	0.025 (2)
C8	0.083 (2)	0.094 (3)	0.078 (2)	0.0131 (19)	0.0467 (18)	0.0042 (18)
C19	0.0695 (18)	0.125 (3)	0.0500 (16)	0.014 (2)	0.0121 (14)	0.0124 (18)
C17	0.110 (3)	0.094 (3)	0.0478 (16)	0.016 (2)	0.0330 (17)	0.0147 (16)
C21	0.113 (3)	0.089 (2)	0.0569 (18)	0.034 (2)	0.0361 (17)	0.0305 (17)
C18	0.092 (2)	0.138 (4)	0.0473 (17)	0.025 (2)	0.0121 (16)	0.0218 (19)
C22	0.245 (6)	0.113 (4)	0.061 (2)	0.023 (4)	0.035 (3)	0.017 (2)

### Geometric parameters (Å, °)

**Table:**

Cl1—C1	1.757 (3)	C9—C8	1.413 (4)
O1—C20	1.200 (3)	C5—C6	1.362 (4)
C13—C12	1.371 (3)	C5—H5	0.9300
C13—C10	1.440 (3)	C16—C17	1.373 (5)
C13—C20	1.454 (3)	C16—C15	1.377 (4)
N2—C12	1.359 (3)	C16—H16	0.9300
N2—C11	1.388 (3)	C15—H15	0.9300
N2—C14	1.430 (3)	C7—C8	1.350 (5)
N1—C1	1.293 (3)	C7—C6	1.392 (5)
N1—C9	1.376 (4)	C7—H7	0.9300
C3—C2	1.366 (3)	C6—H6	0.9300
C3—C4	1.411 (3)	C8—H8	0.9300
C3—H3	0.9300	C19—C18	1.383 (4)
O2—C20	1.339 (3)	C19—H19	0.9300
O2—C21	1.446 (3)	C17—C18	1.354 (5)
C12—H12	0.9300	C17—H17	0.9300
C1—C2	1.420 (3)	C21—C22	1.451 (5)
C14—C19	1.373 (4)	C21—H21A	0.9700
C14—C15	1.375 (4)	C21—H21B	0.9700
C10—C11	1.357 (3)	C18—H18	0.9300
C10—C2	1.477 (3)	C22—H22A	0.9600
C11—H11	0.9300	C22—H22B	0.9600
C4—C9	1.405 (4)	C22—H22C	0.9600
C4—C5	1.419 (4)		
			
C12—C13—C10	107.2 (2)	C6—C5—H5	120.1
C12—C13—C20	123.2 (2)	C4—C5—H5	120.1
C10—C13—C20	129.5 (2)	C17—C16—C15	120.4 (3)
C12—N2—C11	108.49 (19)	C17—C16—H16	119.8
C12—N2—C14	126.1 (2)	C15—C16—H16	119.8
C11—N2—C14	125.3 (2)	C14—C15—C16	120.0 (3)
C1—N1—C9	116.8 (2)	C14—C15—H15	120.0
C2—C3—C4	120.8 (2)	C16—C15—H15	120.0
C2—C3—H3	119.6	C8—C7—C6	121.4 (3)
C4—C3—H3	119.6	C8—C7—H7	119.3
C20—O2—C21	117.9 (2)	C6—C7—H7	119.3
O1—C20—O2	122.2 (2)	C5—C6—C7	120.4 (3)
O1—C20—C13	125.2 (2)	C5—C6—H6	119.8
O2—C20—C13	112.6 (2)	C7—C6—H6	119.8
N2—C12—C13	108.8 (2)	C7—C8—C9	120.0 (4)
N2—C12—H12	125.6	C7—C8—H8	120.0
C13—C12—H12	125.6	C9—C8—H8	120.0
N1—C1—C2	127.1 (2)	C14—C19—C18	119.8 (3)
N1—C1—Cl1	115.3 (2)	C14—C19—H19	120.1
C2—C1—Cl1	117.60 (18)	C18—C19—H19	120.1
C19—C14—C15	119.5 (2)	C18—C17—C16	119.5 (3)
C19—C14—N2	120.1 (2)	C18—C17—H17	120.2
C15—C14—N2	120.4 (2)	C16—C17—H17	120.2
C11—C10—C13	106.4 (2)	O2—C21—C22	109.1 (3)
C11—C10—C2	125.6 (2)	O2—C21—H21A	109.9
C13—C10—C2	128.0 (2)	C22—C21—H21A	109.9
C10—C11—N2	109.1 (2)	O2—C21—H21B	109.9
C10—C11—H11	125.4	C22—C21—H21B	109.9
N2—C11—H11	125.4	H21A—C21—H21B	108.3
C3—C2—C1	115.4 (2)	C17—C18—C19	120.8 (3)
C3—C2—C10	121.6 (2)	C17—C18—H18	119.6
C1—C2—C10	123.0 (2)	C19—C18—H18	119.6
C9—C4—C3	117.9 (2)	C21—C22—H22A	109.5
C9—C4—C5	119.1 (3)	C21—C22—H22B	109.5
C3—C4—C5	122.9 (3)	H22A—C22—H22B	109.5
N1—C9—C4	121.9 (2)	C21—C22—H22C	109.5
N1—C9—C8	118.9 (3)	H22A—C22—H22C	109.5
C4—C9—C8	119.2 (3)	H22B—C22—H22C	109.5
C6—C5—C4	119.9 (3)		
			
C21—O2—C20—O1	4.1 (4)	Cl1—C1—C2—C10	5.3 (3)
C21—O2—C20—C13	−176.6 (3)	C11—C10—C2—C3	67.9 (4)
C12—C13—C20—O1	2.5 (4)	C13—C10—C2—C3	−111.1 (3)
C10—C13—C20—O1	178.0 (3)	C11—C10—C2—C1	−113.8 (3)
C12—C13—C20—O2	−176.7 (2)	C13—C10—C2—C1	67.2 (4)
C10—C13—C20—O2	−1.3 (4)	C2—C3—C4—C9	0.8 (4)
C11—N2—C12—C13	−0.3 (3)	C2—C3—C4—C5	−177.9 (3)
C14—N2—C12—C13	177.7 (2)	C1—N1—C9—C4	−2.8 (4)
C10—C13—C12—N2	−0.5 (3)	C1—N1—C9—C8	177.4 (3)
C20—C13—C12—N2	175.8 (2)	C3—C4—C9—N1	2.2 (4)
C9—N1—C1—C2	0.4 (4)	C5—C4—C9—N1	−179.1 (3)
C9—N1—C1—Cl1	179.1 (2)	C3—C4—C9—C8	−178.0 (3)
C12—N2—C14—C19	153.2 (3)	C5—C4—C9—C8	0.7 (4)
C11—N2—C14—C19	−29.2 (4)	C9—C4—C5—C6	−0.3 (4)
C12—N2—C14—C15	−27.3 (4)	C3—C4—C5—C6	178.4 (3)
C11—N2—C14—C15	150.3 (3)	C19—C14—C15—C16	0.9 (5)
C12—C13—C10—C11	1.1 (3)	N2—C14—C15—C16	−178.6 (3)
C20—C13—C10—C11	−174.9 (2)	C17—C16—C15—C14	0.5 (5)
C12—C13—C10—C2	−179.8 (2)	C4—C5—C6—C7	−0.1 (5)
C20—C13—C10—C2	4.2 (4)	C8—C7—C6—C5	0.0 (6)
C13—C10—C11—N2	−1.2 (3)	C6—C7—C8—C9	0.5 (6)
C2—C10—C11—N2	179.6 (2)	N1—C9—C8—C7	179.0 (3)
C12—N2—C11—C10	1.0 (3)	C4—C9—C8—C7	−0.9 (5)
C14—N2—C11—C10	−177.0 (2)	C15—C14—C19—C18	−1.9 (5)
C4—C3—C2—C1	−2.9 (4)	N2—C14—C19—C18	177.6 (3)
C4—C3—C2—C10	175.5 (2)	C15—C16—C17—C18	−1.0 (6)
N1—C1—C2—C3	2.4 (4)	C20—O2—C21—C22	139.1 (4)
Cl1—C1—C2—C3	−176.30 (19)	C16—C17—C18—C19	0.0 (6)
N1—C1—C2—C10	−176.0 (3)	C14—C19—C18—C17	1.5 (6)

### Hydrogen-bond geometry (Å, °)

**Table:**

*D*—H···*A*	*D*—H	H···*A*	*D*···*A*	*D*—H···*A*
C12—H12···O1^i^	0.93	2.50	3.383 (3)	159
C15—H15···O1^i^	0.93	2.45	3.275 (4)	148

Symmetry codes: (i) −*x*+1/2, −*y*−1/2, −*z*+1/2.
